# Differentiated thyroid carcinoma presentation may be more aggressive in children and adolescents than in young adults

**DOI:** 10.1186/s13052-018-0455-3

**Published:** 2018-01-17

**Authors:** Giuseppina Zirilli, Laura Cannavò, Francesco Vermiglio, Maria Antonia Violi, Filippo De Luca, Malgorzata Wasniewska

**Affiliations:** 1 Department of Human Pathology of Adulthood and Childhood, University of Messina, Messina, Italy; 2 Unit of Endocrinology, Department of Clinical and Experimental Medicine, University of Messina, Messina, Italy; 3 Dipartimento di Patologia Umana dell’adulto e dell’età evolutiva “G. Barresi”, AOU “G. Martino”, Via Consolare Valeria, 98125 Messina, Italy

**Keywords:** Age-related cancer risk, Histologic features, Metastatic diffusion, Phenotypical expression, Thyroid cancer presentation

## Abstract

**Background:**

The available studies concerning the influence of age on the phenotypical expression of differentiated thyroid carcinoma (DTC) have hitherto compared DTC presentation either between pre-pubertal and pubertal children or between pediatric patients and aged adults; aim of this study was to ascertain for the first time whether presentation of DTC may significantly vary according to age, even within a peculiar study population covering only young patients aged less than 30 years.

**Methods:**

The main clinical, biochemical and pathologic data at DTC diagnosis were retrospectively recorded in 2 selected cohorts including, respectively, 18 children and adolescents aged less than 18 years (Group A) or 45 young adults aged between 20 and 29.8 years (Group B).

**Results:**

The statistical distribution of DTC cases in the different age ranges was found to progressively increase with increasing age; furthermore, the patients of Group A exhibited at diagnosis a more severe clinical involvement and a higher rate of extra-regional metastases; finally, also the association with both autoimmune thyroid diseases (AITDs) and a biochemical hypothyroid pattern was more common in Group A patients.

**Conclusions:**

In a study population younger than 30 years: a) the risk of developing DTC increases with age, achieving its zenith during the 3rd decade of life; b) clinical presentation is more severe in children and adolescents younger than 18 years than in the patients aged between 20 and 30; c) in the cohort of children and adolescents DTC is more often associated with AITDs, which might play some role in conditioning the more aggressive phenotypical presentation of DTC in this patient group.

## Background

Thyroid carcinoma is the most common endocrine tumor, with an overall incidence that has been recently reported to have significantly increased in the past two decades [1, 2]. Thyroid cancer is distinctly more rare in childhood than in adulthood [3]. However, similarly to what has been observed in adults, an increasing incidence in thyroid cancer has been documented even in children and adolescents [1, 4].

Other studies have demonstrated that primary thyroid cancer in pediatric patients differs from primary thyroid carcinoma in adults with respect to both presentation and clinical course [5–7]. In children, in fact, differentiated thyroid carcinoma (DTC) may exhibit, at presentation, a more aggressive phenotype, with a higher rate of extrathyroidal extension and either regional lymph-node or pulmonary metastases [8]. In contrast, in children, the more aggressive clinical presentation is not generally associated with a more severe long-term prognosis [9].

Due to all these differences, which seem to characterize the phenotypical expression of DTC in children and adolescents, the American Thyroid Association has recently prepared specific recommendations for the management of thyroid nodules and DTC in young patients up to 18 years of age [10].

In the present study we have retrospectively compared DTC presentation in two selected patient cohorts including, respectively, either children and adolescents aged less than 18 years or young adults aged between 20 and less than 30 years. Aim of our study was to ascertain whether DTC presentation may significantly vary according to age, even within a peculiar study population covering only young patients aged less than 30 years.

## Materials and methods

### Study population

It consisted of all the 63 patients < 30 years of age with primary thyroid cancer and well-documented histologic diagnosis of DTC, who were identified in our Departments during the period 1999–2016. Therefore, the patients with anaplastic cancer were not taken into consideration for this study.

It has to be clarified that almost the totality of the children and adults with thyroidal disorders who live in the province of Messina are, as a general rule, examined in our Departments. Our series, however, included also a minority of 10 patients living in the province of Reggio Calabria or in other Sicilian districts. In this territory, profound improvements in nutritional iodine status have been recorded over the last 30 years, due to both silent and active iodine prophylaxis programs [11].

### Study design

The data registered at the time of DTC diagnosis were retrospectively collected from medical charts and used for the present study. Such data included: presenting symptoms and findings, thyroid function and autoimmunity tests, imaging procedures (cervical ultrasound US scan, chest radiograph, computed tomography scan) and the cytological findings yielded with fine needle aspiration (FNA).

Post-surgical data on tumor features included size, histologic type, presence of microscopic neoplastic foci in the gland outside of the main nodule, subcapsular or extracapsular infiltration and either regional or distant metastases.

All the above data were evaluated separately in the patients aged less than 18 years (Group A) and in those aged between 20.0 and 29.8 years at the time of DTC diagnosis (Group B).

### Methods

Thyroid function and autoimmunity investigations, which were performed in all the 63 patients, included the measurement of TSH, FT4, thyroid peroxidase and thyroglobulin autoantibody (TPOAb and TGAb, respectively) serum levels. TSH receptor autoantibody (TRAB) serum levels were occasionally measured, at DTC diagnosis, only in the cases with a biochemical hyperthyroid picture.

FT4 (reference range for our laboratory 10.3–24.4 pmol/L) and TSH levels (reference range 0.3–4.5 mIU/L) were measured by radioimmunoassay, whilst TGAb (reference range 0–30 IU/ml), TPOAb (reference range 0–20 IU/ml) and TRAB levels (reference range 0–4 IU/ml) were measured by chemiluminescent immunometric assays [12, 13].

With regard to thyroid function at diagnosis, the patients were classified into the following biochemical groups: 1) euthyroidism (both TSH and FT4 within normal limits); 2) overt hypothyroidism (elevated TSH with low FT4); 3) subclinical hypothyroidism (elevated TSH with normal FT4); 4) overt hyperthyroidism (suppressed TSH with elevated FT4); 5) subclinical hyperthyroidism (suppressed TSH with normal FT4).

Thyroid US was performed with a high resolution 7.5 MHz linear transducer.

The patients with a hypoechogenic gland pattern at US compatible with autoimmune thyroid diseases (AITDs) and positivity for serum TPOAbs and/or TGAbs were considered as having an association between DTC and Hashimoto’s thyroiditis (HT). Those with hypoechogenic US pattern, biochemical hyperthyroidism and positivity for serum TRABs were considered as having an association between DTC and Graves’ disease (GD).

FNA biopsy and interpretation of the specimen was performed by a skilled staff of our Department. Cytological findings were classified according to Bethesda system [14].

Pathologic staging of DTC at diagnosis was assessed according to the tumor-node-metastasis classification system adopted by the 7th American Joint Committee on Cancer [10]. According to this staging system, patients with DTC should be stratified into 3 different risk levels (low, intermediate or high), based on the possible evidence of either regional lymph-node invasion or distant metastases [10]. In particular, pediatric risk level is defined as low in the cases with disease grossly confined to the gland and no extrathyroidal extension, intermediate in the cases with regional lymph-node metastases and high in the cases with extra-regional distant metastases [10].

The lesions which were referred to as “incidentaloma” were small and clinically non-palpable and were incidentally discovered at US [15].

### Statistical analysis

Patient age was expressed as median and range.

Comparisons between the 2 selected groups were performed by Chi-square test. The level of significance was set at 0.05.

## Results

### Clinical data at DTC diagnosis

In the overall series of 63 young patients < 30 years, who were identified in our Departments during the period 1999–2016 to be affected by a primary DTC, the female: male ratio was 2.7 (73.0% females). A preponderance of females was recorded in both patient groups: 72.5% in Group A vs 73.4% in Group B.

Patients’ median age was 24.0 years (range 9.7–29.8). None of the 63 patients was aged between 18 and 19.9 years.

The statistical distribution of DTC cases in the different age ranges was found to progressively increase with increasing age: from 1.6% before age 10 years to 11.1% at 10–14 years, 15.9% at 14.1–18 years, 22.2% at 20–24 years, 49.2% at 24.1–29.8 years. Therefore, the majority of DTC cases (71.4%) belonged to Group B, whose median age was 26.0 years (range 20.0–29.8) vs 15.7 (range 9.7–17.9) in Group A.

In Group A individuals a common clinical finding at diagnosis was cervical lymphadenopathy, which was observed in 38.8% of cases (vs only 2.2% of cases in Group B, *p* = 0.0001). In contrast, at presentation, an incidentaloma was significantly more common in the patients of Group B: 66.6% of cases vs 33.3% in Group A, *p* = 0.009. An anterior neck mass was found in 38.8% of Group A patients and in 24.4% of Group B patients.

### Thyroid function patterns and association with AITDs at DTC diagnosis

At diagnosis, almost the totality of patients (88.9%) were euthyroid. The prevalence of euthyroidism at presentation was significantly higher in Group B, whilst the prevalence of patients with a biochemical picture of overt hypothyroidism was significantly higher in Group A (Table 1). A picture of subclinical hypothyroidism was detected in only one patient of Group B and in none of Group A patients. The prevalence of overt hyperthyroidism was not significantly different in the 2 groups (Table 1). No patients of both groups exhibited a biochemical picture of subclinical hyperthyroidism.

**Table 1:** Main biochemical, clinical, cytological and histologic findings at diagnosis of primary differentiated thyroid cancer in 63 patients less than 30 years: comparisons between children aged from 9.7 and 17.9 years (Group A) and young adults aged from 20.0 and 29.8 years (Group B)

	Group A (*n* = 18)	Group B (*n* = 45)	*p*
Biochemical picture			
Euthyroidism	72.3%	95.6%	0.03
Overt hypothyroidism	16.6%	0%	0.005
Subclinical hypothyroidism	0%	2.2%	0.52
Overt hyperthyroidism	11.1%	2.2%	0.10
Clinical picture			
Association with AITDs^a^	33.3%	8.9%	0.02
Cytological stages^b^			
III	22.2%	22.2%	1.00.
IV	27.8%	33.3%	0.67
V	27.8%	28.9%	0.93
VI	22.2%	15.6%	0.53
Tumor histology			
Papillary carcinoma	55.6%	66.7%	0.40
Papillary follicular variant	33.3%	31.1%	0.86
Follicular carcinoma	11.1%	2.2%	0.13
Risk levels^c^			
Low	55.5%	75.6%	0.26
Intermediate	27.8%	22.2%	0.73
High	16.7%	2.2%	0.03

^a^Autoimmune thyroid diseases

^b^According to the Bethesda system (Reference No. 14)

^c^According to the staging system described by Francis et al. (Reference No.10)

The association with AITDs, either HT or GD, was found to be, at presentation, significantly more common in Group A patients (Table 1).

### Cytological findings

The distribution of cytological stages was not significantly different among the patients of both groups (Table 1).

### Histologic characteristics and risk levels of DTC, according to its extension at diagnosis

In the entire study population, the most common histological subtype was papillary carcinoma (in 63.5% of cases), followed by follicular variant of papillary carcinoma (in 31.7%) and follicular cancer (in 4.8% of cases). No cases with medullary carcinoma were detected in the present series.

The prevalence of these histologic subtypes did not significantly differ in the 2 patient groups (Table 1).

At diagnosis, the prevalence of cases with either low or intermediate risk levels was not significantly different in the 2 patient groups, whereas the prevalence of individuals with high risk levels was significantly more elevated in Group A (Table 1).

## Discussion

The available studies aiming to investigate the influence of age on the phenotypical expression of DTC have hitherto compared DTC presentation and course either between pre-pubertal children and adolescents [16] or between pediatric patients and aged adults [17]. Therefore, to the best of our knowledge, this is the first study aiming to ascertain whether DTC presentation may significantly vary according to age, even within a selected study population covering only young patients aged less than thirty years.

According to our results we can infer that the phenotypical expression of DTC in children and adolescents is really more aggressive, even when compared with that observed in young adults aged between twenty and thirty years. In fact, the patients of our series belonging to Group A exhibited, at diagnosis, a more severe clinical involvement, as suggested by the higher prevalence of both lymphadenopathies and distant parenchymal metastases.

The underlying mechanisms, which might be involved in the pathophysiology of the more extensive DTC presentation in children and adolescents than in young adults, are not clear, although a predisposing role of concomitant AITDs might be hypothesized to explain, at least partially, such an age-related clinical peculiarity.

In fact, it has to be considered that, in the present study, an association with AITDs was found to be more common in the patients of Group A, who exhibited also, when compared to those of Group B, an increased prevalence of biochemical thyroid function alterations compatible with overt hypothyroidism.

Interestingly, the complex relationships between thyroid autoimmunity and DTC have been just recently focused and it was hypothesized that HT could exert a promoting effect on DTC growth, either directly or through a secondary chronic elevation of TSH serum levels [18]. The most recent views on this topic, therefore, support the idea that both children and adults with HT may be potentially more incline to the risk of DTC [19–23]. Such risk seems to increase with increasing serum levels of TSH and thyroid autoantibodies [22].

The association with HT has been reported to play some predisposing role even in conditioning an increased risk of DTC metastases, especially in the patients with secondary hypothyroidism [24].

Even acute suppurative thyroiditis has been sporadically reported to be possibly associated with DTC and to represent, therefore, a potential pre-cancerous condition [25].

On the other hand, if an underlying gland inflammation may facilitate the development and diffusion of DTC, it is also true that an increased attention to the thyroid gland can facilitate an earlier diagnosis of cancer. This might explain the apparent paradox that children and adolescents with DTC have generally an excellent long-term prognosis, although cancer in this age group often presents with more advanced disease [16].

Finally, a further inference which could be suggested by the analysis of our results is that, in the context of a population aged less than thirty years, the risk of developing a DTC is almost negligible during the first ten years of life and increases during the second decade, achieving its zenith during the third decade. A very similar age-related trend had been already observed in both USA and Europe during the past years [3, 26], which reinforces the significance of our epidemiological findings.

## Conclusions

In a study population younger than 30 years: a) the risk of developing DTC increases with age, achieving its zenith during the 3rd decade of life; b) clinical presentation is more severe in children and adolescents younger than 18 years than in the patients aged between 20 and 30; c) in the cohort of children and adolescents DTC is more often associated with both AITDs and HT-related biochemical hypothyroidism, which might play some role in conditioning the more aggressive clinical presentation of DTC in this patient group; d) biopsy results and histologic cancer subtypes do not significantly differ in the two patient groups.

## Abbreviations

AITDs: autoimmune thyroid diseases
DTC: differentiated thyroid carcinoma
FNA: fine needle aspiration
GD: Graves’ disease
HT: Hashimoto’s thyroiditis
TGAb: thyroglobulin autoantibody
TPOAb: thyroid peroxidase autoantibody
TRAB: TSH receptor autoantibody

## References

[1] Vergamini LB, Frazier AL, Abrantes FL, Ribeiro KB, Rodriguez-Galindo C (2014). Increase in the incidence of differentiated thyroid carcinoma in children, adolescents, and young adults: a population-based study. J Pediatr.

[2] Russo M, Malandrino P, Moleti M, D'Angelo A, Tavarelli M, Sapuppo G (2017). Thyroid cancer in the pediatric age in Sicily: influence of the volcanic environment. Anticancer Res.

[3] Siegel DA, King J, Tai E, Buchanan N, Ajani UA (2014). Cancer incidence rates and trends among children and adolescents in the United States, 2001-2009. Pediatrics.

[4] McNally RJ, Blakey K, James PW, Gomez Pozo B, Basta NO, Hale J (2012). Increasing incidence of thyroid cancer in great Britain, 1976-2005: age-period-cohort analysis. Eur J Epidemiol.

[5] Grigsby PW, Gal-or A, Michalski JM, Doherty GM (2002). Childhood and adolescent thyroid carcinoma. Cancer.

[6] Park S, Jeong JS, Ryu HR, Lee CR, Park JH, Kang SW (2013). Differentiated thyroid carcinoma of children and adolescents: 27-year experience in the yonsei university health system. J Korean Med Sci.

[7] Hacıhamdioğlu B, Oçal G, Berberoğlu M, Savaş Erdeve S, Camtosun E, Kocaay P (2014). the evaluation of thyroid carcinoma in childhood and concomitance of autoimmune thyroid disorders. J Pediatr Endocrinol Metab.

[8] LaFranchi SH (2015). Inaugural management guidelines for children with thyroid nodules and differentiated thyroid cancer: children are not small adults. Thyroid.

[9] Markovina S, Grigsby PW, Schwarz JK, DeWees T, Moley JF, Siegel BA (2014). Treatment approach, surveillance, and outcome of well-differentiated thyroid cancer in childhood and adolescence. Thyroid.

[10] Francis GL, Waguespack SG, Bauer AJ, Angelos P, Benvenga S, Cerutti JM (2015). management guidelines for children with thyroid nodules and differentiated thyroid cancer. Thyroid.

[11] Moleti M, Sturniolo G, Trimarchi F, Vermiglio F (2016). The changing phenotype of iodine deficiency disorders: a review of thirty-five years of research in north-eastern Sicily. Ann Ist Super Sanita.

[12] Wasniewska M, Corrias A, Arrigo T, Lombardo F, Salerno M, Mussa A (2010). Frequency of Hashimoto's thyroiditis antecedents in the history of children and adolescents with graves' disease. Horm Res Paediatr..

[13] Wasniewska M, Corrias A, Salerno M, Lombardo F, Aversa T, Mussa A (2012). Outcomes of children with hashitoxicosis. Horm Res Paediatr.

[14] Cibas ES, Ali SZ, NCI thyroid FNA state of the science conference (2009). The Bethesda system for reporting thyroid cytopathology. Am J Clin Pathol.

[15] Singh S, Singh A, Khanna AK (2012). Thyroid incidentaloma. Indian J Surg Oncol.

[16] Lazar L, Lebenthal Y, Steinmetz A, Yackobovitch-Gavan M, Phillip M (2009). Differentiated thyroid carcinoma in pediatric patients: comparison of presentation and course between pre-pubertal children and adolescents. J Pediatr.

[17] Solymosi T, Lukacs Toth G, Budai L, Gal I (2016). The clinical and pathological presentation of thyroid nodules in children and the comparison with adult population: experience of a single institution. Int J Endocrinol.

[18] Boi F, Pani F, Mariotti S (2017). Thyroid autoimmunity and thyroid cancer: review focused on cytological studies. Eur Thyroid J.

[19] Gasbarri A, Sciacchitano S, Marasco A, Papotti M, Di Napoli A, Marzullo A (2004). Detection and molecular characterisation of thyroid cancer precursor lesions in a specific subset of Hashimoto's thyroiditis. Br J Cancer.

[20] Corrias A, Cassio A, Weber G, Mussa A, Wasniewska M, Rapa A (2008). Thyroid nodules and cancer in children and adolescents affected by autoimmune thyroiditis. Arch Pediatr Adolesc Med.

[21] Fiore E, Rago T, Latrofa F, Provenzale MA, Piaggi P, Delitala A (2011). Hashimoto's thyroiditis is associated with papillary thyroid carcinoma: role of TSH and of treatment with L-thyroxine. Endocr Relat Cancer.

[22] Boi F, Minerba L, Lai ML, Marziani B, Figus B, Spanu F (2013). both thyroid autoimmunity and increased serum TSH are independent risk factors for malignancy in patients with thyroid nodules. J Endocrinol Investig.

[23] Lee JH, Kim Y, Choi JW, Kim YS (2013). The association between papillary thyroid carcinoma and histologically proven Hashimoto's thyroiditis: a meta-analysis. Eur J Endocrinol.

[24] Paulson LM, Shindo ML, Schuff KG (2012). Role of chronic lymphocytic thyroiditis in central node metastasis of papillary thyroid carcinoma. Otolaryngol Head Neck Surg.

[25] Crisafulli G, Wasniewska M, Ascenti G, Rulli I, Zirilli G, Aversa T (2008). Acute suppurative thyroiditis disclosing diagnosis of thyroid cancer in a boy. J Endocrinol Investig.

[26] Steliarova-Foucher E, Stiller CA, Pukkala E, Lacour B, Plesko I, Parkin DM (2006). Thyroid cancer incidence and survival among European children and adolescents (1978-1997): report from the automated childhood cancer information system project. Eur J Cancer.

